# Socioeconomic inequalities in high-risk fertility behaviors over time in Ethiopia

**DOI:** 10.1371/journal.pone.0313028

**Published:** 2024-12-31

**Authors:** Melash Belachew Asresie, Michael Ekholuenetale, Kedir Y. Ahmed, Sabuj Kanti Mistry, Navira Chandio, Kingsley Agho, Gedefaw Abeje Fekadu, Amit Arora

**Affiliations:** 1 Department of Reproductive Health and Population Studies, School of Public Health, College of Medicine and Health Sciences, Bahir Dar University, Bahir Dar, Ethiopia; 2 Facility of Science and Health, School of Health and Care Professions, University of Portsmouth, Portsmouth, United Kingdom; 3 Department of Epidemiology and Medical Statistics, Faculty of Public Health, College of Medicine, University of Ibadan, Ibadan, Nigeria; 4 Rural Health Research Institute, Charles Sturt University, Orange, NSW, Australia; 5 School of Population Health, University of New South Wales, Sydney, Australia; 6 Department of Public Health, Daffodil International University, Dhaka, Bangladesh; 7 School of Health Sciences, Western Sydney University, Campbelltown Campus, Penrith, NSW, Australia; 8 Translational Health Research Institute, Western Sydney University, Campbelltown Campus, Penrith, NSW, Australia; 9 Health Equity Laboratory, Campbelltown, NSW, Australia; 10 Faculty of Health Sciences, University of Johannesburg, Johannesburg, South Africa; 11 Discipline of Child and Adolescent Health, The Children’s Hospital at Westmead Clinical School, Faculty of Medicine and Health, The University of Sydney, Westmead, NSW, Australia; 12 Oral Health Services, Sydney Local Health District and Sydney Dental Hospital, NSW Health, Surry Hills, NSW, Australia; University of Salamanca, SPAIN

## Abstract

**Introduction:**

High-risk fertility behaviors (HRFB), including short birth intervals, early or late childbearing age, and high parity, are associated with adverse pregnancy outcomes. Understanding the importance of socioeconomic disparity in HRFB and the factors influencing this disparity is essential to improve maternal and child survival, Accordingly, this study investigated socioeconomic inequalities in HRFB over time and its contributing factors.

**Methods:**

We included a total weighted sample of 11,163 and 5,527 women aged 15 to 49 years from the 2005 and 2019 Ethiopia Demographic and Health Surveys, respectively. Erreygers Concentration index (ECI) and curve, along with Erreygers normalized decomposition analysis, were used to examine socioeconomic-related inequalities in HFRB and identify contributing factors to these inequalities.

**Results:**

The study showed that the concentration curve for HFRB remained above the equality line over time, indicating a disproportionate concentration among socioeconomically disadvantaged individuals. In 2005, the pro-poor ECI was -0.0682; in 2019, it was -0.2634, indicating that pro-poor inequality has widened. Educational status (10% in 2005 and 28% in 2019), place of birth (7% in 2005 and 28% in 2019), religion (16% in 2005 and 4% in 2019), and region (9% in 2005 and 3% in 2019) contributed to the observed pro-poor inequality. In 2019, contraceptive use (12%) and wealth index (15%) emerged as additional factors explaining HRFB inequality.

**Conclusion:**

Our findings revealed the disproportional concentration of HRFB among socioeconomically disadvantaged women in Ethiopia, with a widening disparity between 2005 and 2019. Future interventions to address the effect of socioeconomic disadvantage on HRFB should prioritize women with low or no formal education, those who give birth at home, and those who do not use contraceptives.

## Introduction

Although the global population growth is projected to be declining, the sub-Saharan Africa (SSA) region has not exhibited a slowdown [1]. The SSA’s population is projected to reach 1.2 billion by 2025, and addressing population-related challenges in the region becomes increasingly urgent [2]. In addition, maternal and newborn mortality rates have decreased globally, but disparities persist, particularly in SSA [3, 4]. Furthermore, adverse pregnancy outcomes are still prevalent, particularly in low and middle-income countries (LMICs) [5]. This is because of the high-risk fertility behavior (HRFB) including short birth intervals, early or late childbearing age, and high parity in SSA countries, which largely persist in the region [6–10].

HRFB significantly impacts maternal health during pregnancy and childbirth, leading to complications like obstetric hemorrhage, miscarriage, hypertensive disorders, and maternal mortality [6, 10]. HRFB is linked to an elevated risk of obstetric complications because of the immaturity of the mother’s reproductive system for younger women. This condition can lead to maternal depletion syndrome, and nutritional deficiencies and increases the likelihood of preterm birth, congenital anomalies, low birth weight, as well as perinatal and neonatal mortality [6, 11, 12]. Furthermore, these adverse outcomes not only impact the immediate pregnancy outcomes but have long-term consequences for maternal health and subsequent pregnancies [7, 9]. While there has been a global decrease in the burden of HRFB, it continues to pose a significant public health concern, particularly in regions such as SSA, including Ethiopia. Recent data suggests that approximately 58% of women in East Africa are involved in at least one type of HRFB [13].

Ethiopia, one of the largest countries in SSA, has experienced rapid population growth and high fertility rates [14]. Even though there has been significant progress in reducing maternal and neonatal mortality, Ethiopia still lags behind national targets and faces increasing neonatal mortality rates and adverse pregnancy outcomes recently [15–17]. This is because of the high prevalence of HRFB in the country, with three-fourths of women experiencing at least one type of HRFB [9, 18]. In recent decades, Ethiopia has implemented various strategies to address the burden of HRFB. These initiatives include developing population policies, enforcing regulations to combat early marriage, allowing legal termination of pregnancy for women under 18 years of age, and establishing family planning programs to reduce population growth rates while enhancing maternal, child, and community health [14, 19]. Furthermore, in 2005, Ethiopia introduced the Health Extension Program, which operates at the grassroots level to provide free maternal health services, particularly targeting rural and underserved areas to improve access to essential healthcare services [20]. Consequently, contraceptive coverage has increased from 7% in 2000 to 41% in 2019, and the fertility rate has declined from 6 births per woman in 2000 to 4 births per woman in 2019 [7]. However, 22% of married women face an unmet need for family planning and have undergone at least one unwanted childbirth [21]. Moreover, the prevalence of HRFB has remained consistently high and unchanged over the last two decades [18, 22], possibly due to disparities in access to and utilization of reproductive healthcare services [23].

Health inequality is the most serious threat to global public health, defined as a systematic disparity in health across individuals and groups with greater or lesser advantages [24, 25]. The situation is particularly worse in low-income countries [26]. Consequently, addressing health inequality has emerged as a top priority on the World Health Organization’s agenda [27]. Health inequality is also an agenda of Sustainable Development Goal 10 (SDG 10), which aims to reduce inequality in health outcomes within and among nations [28]. Even though health inequality remains a global and many country health policy agenda including Ethiopia, progress has been inadequate towards achieving this goal [29]. A study in Ethiopia has shown that there are significant inequalities in maternal and child health service access based on socioeconomic status [30]. Moreover, socioeconomic disparities in maternal health services and contraceptive use have been found within the country, with individuals of higher socioeconomic status more likely to utilize them [31, 32]. Furthermore, access to healthcare and health information, autonomy in health-seeking services, nutritional intake, and lifestyle decisions depend on socioeconomic status [33].

Previous studies in Ethiopia examined the magnitude and determinants of HRFB reporting the odds ratio as a measure of association [18, 22]. However, the determinants of HRFB might be differently distributed depending on the mothers’ socioeconomic status. Exploring the impact of socioeconomic status on the distribution of determinants of HRFB can provide valuable insights into existing inequalities. Hence, the conventional concentration index better allows inequalities to be estimated across the whole population (i.e., in a cumulative share of women ranked by wealth index) using a single metric [34]. Moreover, while the concentration index can be decomposed to a range of variables that drive the socioeconomic-related inequality, this may not be true for the odds ratio primarily measures the association strength [35]. However, to our knowledge, no prior study has investigated socioeconomic disparities in HRFB over time. Hence, this study aimed to examine changes in socioeconomic-related inequality in HRFB and its contributing factors. It employed data from the 2005 and 2019 Ethiopian Demographic and Health Surveys (EDHS), utilizing Erreygers normalized decomposition analysis. It is a method used to quantify and understand socioeconomic inequality within a population, particularly in healthcare contexts. It examines how various factors contribute to inequality, providing insights for policy interventions aimed at reducing disparities [36].

## Methods

### Data source and study design

This cross-sectional study was based on the secondary analysis of the 2005 EDHS and the 2019 mini-EDHS datasets. The EDHS is a community-based cross-sectional survey designed to provide national, regional, and residential representation to assess maternal and child health indicators. Data collection for the 2005 survey was between April 27 and August 30, 2005, while for the 2019 survey, it took place between March 21 and June 28, 2019. Ethiopia, located in sub-Saharan Africa, shares borders with Eritrea to the north, Djibouti to the northeast, Sudan to the west, Somalia to the east and northeast, and Kenya to the south. Ethiopia was administratively divided into nine regions and two cities during the data-collecting period [7, 37].

### Study population and sample size

The target population for the EDHS comprised all reproductive-age women (15 to 49 years). However, the specific objective of this study was to examine HRFB among women who had given birth within the five years preceding the survey. Hence, the final analysis contained a weighted sample of 11,163 and 5,527 reproductive-age women who had given birth in the five years preceding the survey, in 2005 and 2019, respectively. These women were asked about their age at birth, birth interval, and birth order to understand their fertility behaviors [7, 35].

### Sampling technique and procedure

**Fig 1:**
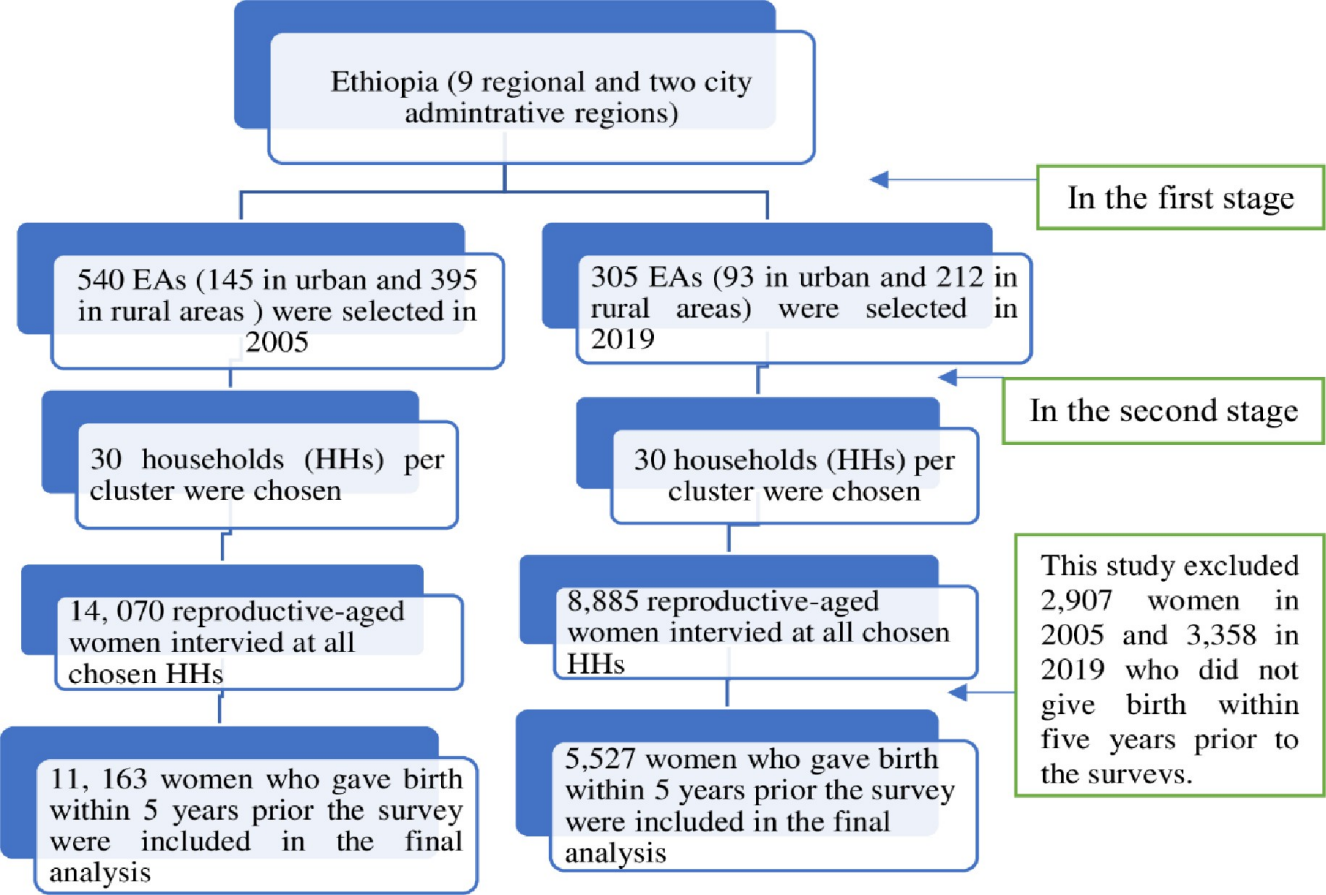
Schematic presentation of the sampling process for women who gave birth within five years preceding the 2005 and 2019 EDHS to study socioeconomic inequality in HRFB.

For the 2005 EDHS, the sampling frame was based on the 1994 Ethiopian Population and Housing Census (PHC). For the 2019 EDHS, the sampling frame relied on the planned 2019 Ethiopian PHC, which was intended to be conducted by the Ethiopia Central Statistics Agency. The EDHS utilized a two-stage stratified cluster sampling method. Initially, each region was stratified into urban and rural areas. These areas are then further subdivided into zones, then districts, and finally into smaller, manageable units called enumeration areas (EAs), yielding 21 sampling strata. Then clusters within each stratum are selected, and households within the chosen clusters are selected for data collection. In the first stage of sampling, 540 enumeration areas (EAs) (145 urban and 395 rural) were selected for the 2005 survey, and 305 EAs (93 in urban and 212 in rural areas) were selected for the 2019 survey. In the second stage of sampling, 30 households per cluster were chosen using an equal-probability systematic selection approach within each enumeration area. All reproductive-aged women (15–49 years) who were either permanent residents of the selected households or visitors who slept in the households the night before the survey were eligible to be interviewed to assess maternal and child health-related indicators (Fig 1). The Ministry of Health (MoH) requested the survey, and the Ethiopian Public Health Institute (EPHI) conducted it [7, 35].

### Measurements

Socioeconomic inequality in avoidable HRFB was the outcome variable among women aged 15–49 years. HRFB was computed based on the four parameters: (I) mothers aged <18 years at the time of delivery; (II) mothers aged >34 years at the time of delivery; (III) mothers of a child born after a short birth interval (<24 months); and (IV) mothers of high parities (>3 children). If a woman experienced one of the above high-risk factors, she was defined as having experienced HRFB, coded as 1 or “yes,” otherwise 0 or "no" [7, 35]. The socioeconomic inequality of HRFB can be expressed as the covariance between HRFB and the fractional rank in the living standards (wealth index). The wealth index is used to measure the socioeconomic status of the household based on their assets. A wealth index was constructed using principal components analysis, based on household assets. The asset scores were standardized to a normal distribution with a mean of zero and a standard deviation of one. Each household received a standardized score based on asset possession, and these scores were summed to rank individuals. The sample was then divided into quintiles, each comprising 20% of the population, and used to classify as pro-poor or pro-rich or as having inequality problems. The lowest quintile represented the poorest households, followed by the poor, middle-class, and the top 40% for the rich and richest households. After conducting a thorough review of various literature sources [38] and assessing the availability of variables in both the 2005 and 2019 EDHSs, the following variables were identified as key explanatory variables for decomposition analysis: religion, marital status, educational status, sex of the household head, wealth index rank, residence, region, sex of children, Place of birth, and contraceptive use. These variables were deemed essential for understanding and analyzing demographic and health outcome changes over time and across different population groups.

### Data management and analysis

The samples were weighted to correct disproportionate sampling and non-response throughout the analysis using STATA software version 15.1. The “svyset” command was also applied to handle the effect of complex sample surveys in the EDHS.

The concentration index (CI) was calculated to measure the extent of socioeconomic-related inequalities in HRFB. The CI is defined as twice the space enclosed by the concentration curve, denoted as L(p), and the equity line at 45 degrees extends from the bottom corner to the top right [39]. In this case, it provides the distribution of HRFB across wealth status which ranges from the poorest to the richest. The equity line, ranging from -1 to +1, indicates the direction of the relationship between HRFB and the distribution of living standards (wealth status). When the CI value is negative, it shows that the health variable, in this case, HRFB is predominantly concentrated among the more disadvantageous (pro-poor) individuals. When CI = -1, it indicated that the poorest people had all of the health variables, in this case, HRFB. Conversely, when the CI value is positive, it indicates that the health variable is concentrated among the more advantaged (pro-rich) individuals. When the CI = 1, it displayed that the richest people had all of the health variables (HRFB). When the CI = 0, the distribution was proportionate; in this case, when there is no socioeconomic-related inequality, the concentration curve lies at a 45-degree line (the line of perfect equality) [34, 39]. The CI of covariances (socioeconomic status) between the health variables (HRFB) can be expressed as:


$$C=\frac{2}{\mu }\;cov\;\left(h,r\right)$$

Where C indicates the CI, μ is the mean health variable (in this case, the percentage of HRFB), h is the health variable (HRFB), and *r* Each woman represents the cumulative percentage across the population after ranking HRFB by socioeconomic status. For unbounded variables, the index ranges from -1 to 1, whereas for bounded variables, it ranges from μ-1 to 1-μ [40].

However, the current study’s outcome variable was binary coded as 1 for experienced HRFB and 0 for not experienced HRFB. The boundaries of the concentration index (C) are determined based on the mean of the outcome variable (μ) and do not range from -1 to 1. Instead, the Erreygers normalized concentration index (ECI) [36], a modified version of the concentration index, was employed in this study. The boundaries of ECI range from μ-1 (lower bound) to 1-μ (upper bound). Mathematically, the ECI can be defined as:


ECI=4*μ*CI(Y)

Where ECI is Erreygers concentration index, CI (y) is the generalized concentration index, and μ is the mean of the health variables (HRFB).

The concertation curve is used to graphically depict the socioeconomic-related inequalities in HRFB. The curves plot the cumulative percentage of HRFB (y-axis) against the cumulative share of the population ranked by living standards beginning with the poorest and ending with the richest (x-axis) [39] to socioeconomic-related inequality in HRFB. When there’s no socioeconomic-related inequality, the concentration index (CI) is zero. This means that if an individual, irrespective of her living standards, has the same HRFB value, the concertation curve lies at a 45-degree line, running from the bottom left-hand corner to the top right-hand corner, termed the "line of equality." The concentration curve lying above and below the equality line (45%) indicates that the health variable is disproportionately concentrated between poor (pro-poor) or ECI<0) and rich (pro-rich or ECI>0, respectively [34].

Visual inspection alone of a concentration curve compared to the line of equality isn’t enough to determine if dominance is statistically significant. Hence, a dominance test was conducted to assess the significance of the difference between the concentration curve and the line of equality (45-degree diagonal line). A 95% confidence interval was calculated and p-values less than 0.05 were used to declare statistical significance [39].

Furthermore, to determine the relative contribution of various determinants to socioeconomic-related disparity in HRFB, a decomposition of the ECI was undertaken [34, 39, 40]. For any linear additive regression model of health outcome (y) [39], such as

$$y=\alpha +\sum_{k}\beta_{k}X_{k}+\in$$

The concertation index for y CI is given as:

$$C=\sum_{k}\left(\frac{\beta_{k}{\bar{X}}_{k}}{\mu }\right)C_{k}+\frac{gc_{\in }}{\mu }$$

Where “y” is the health outcome variable this case socioeconomic-related inequality of HRFB, *X*_*K*_ is a set of the socioeconomic determinants of the health outcome, α is the intercept, *β*_*k*_ is the coefficient of *X*_*K*_, μ is the mean of y, ${\bar{X}}_{K}$ is the mean of *X*_*K*_, *C*_*k*_ is the concertation index for *X*_*k*_ (defined analogously to C), *gc*_*∈*_ is the generalized concertation index for the error term/residual term (∈), $\frac{\beta_{k}{\bar{X}}_{k}}{\mu }$ is the elasticity of y (HRFB) with respect to ${\bar{X}}_{K}$ which is the impact of each determinant on the HRDB [35, 40]. The residual (∈), reflects the inequality in HRFB that cannot be explained by systematic variation across income groups in the *X*_*K*_, which would approach zero for a well specific model.

### Ethical considerations

After a request letter submitted to the Demographic and Health Surveys (DHS) program, the International Review Board (ICF) of DHS program data archivists authorized us to download the data set and use these for this study. The datasets were not shared or passed on to other organizations or individuals, and they were kept strictly confidential. The primary data were collected by national and international ethical norms.

## Results

### Participant characteristics

The study included 11,163 women in 2005 and 5,525 in 2019 EDHS. The mean age of women was 29.3 years in 2005 and 29.0 years in 2019, with a standard error (SE) of 0.1 in both surveys. In EDHS 2005, around 79% of women had no formal education, compared to 54% in 2019. In 2005, over 93% of women lived in rural areas, compared to 75% in 2019. Almost 40% of the women in both surveys were from the Oromia region. In 2005, about 6% of women delivered at health facilities, while in 2019, it was 49%. Almost 15% of women in 2005 and 18% of women in 2019 were from the wealthiest households (Table 1).

**Table 1:** Characteristics of women who gave birth within the last five years preceding EDHS 2005 (n = 11,163) and 2019 (n = 5,527), Ethiopia.

Variables	2005	2019
	Weighted frequencies n (%)	Weighted frequencies n (%)
Education status		
No education	8,838 (79.2)	2,662 (53.6)
Primary	1,855 (16.6)	1,956 (35.4)
Secondary	427 (3.8)	415 (7.5)
Higher	43 (0.4)	194 (3.5)
Marital status		
Not in union	645 (5.8)	302 (5.5)
In union	10,518 (94.2)	5,225 (94.5)
Sex of household head		
Male	9,944 (89.1)	4,776 (86.4)
Female	1,219 (10.9)	751 (13.6)
Religion		
Orthodox	4,674 (41.9)	1,860 (33.7)
Protestant	2,217 (19.9)	1,460 (26.4)
Muslim	3,875 (34.7)	2,101 (38.0)
Other	397 (3.5)	106 (1.9)
Residence		
Rural	10,348 (92.7)	4,160 (75.3)
Urban	815 (7.3)	1,367 (24.7)
Region		
Tigray	698 (6.3)	371 (6.7)
Amhara	2,621 (23.5)	1,050 (19.0)
Oromia	4,411 (39.5)	2,211 (40.0)
Somali	477 (4.3)	409 (7.4)
SNNPR	2,500 (22.4)	1,106 (20.0)
City Administrative	191 (1.7)	186 (3.4)
Other	265(2.4)	194 (3.5)
Place of birth		
Home	10,528 (94.3)	2,842 (51.4)
Health facility	635 (5.7)	2,685 (48.6)
Sex of the baby		
Male	5,723 (51.3)	2,842 (51.4)
Female	5,440 (48.7)	2,685 (48.6)
Contraceptive use		
No	9,853 (88.3)	3,312 (59.9)
Yes	1,310 (11.7)	2,215 (40.1)
Wealth index		
Poorest	2,440 (21.9)	1,321 (23.9)
Poorer	2,356 (21.1)	1,198 (21.7)
Middle	2,486 (22.3)	1,043 (18.9)
Richer	2,221 (19.9)	960 (17.3)
Richest	1,660 (14.9)	1,005 (18.2)

### High-risk fertility behaviors

**Fig 2:**
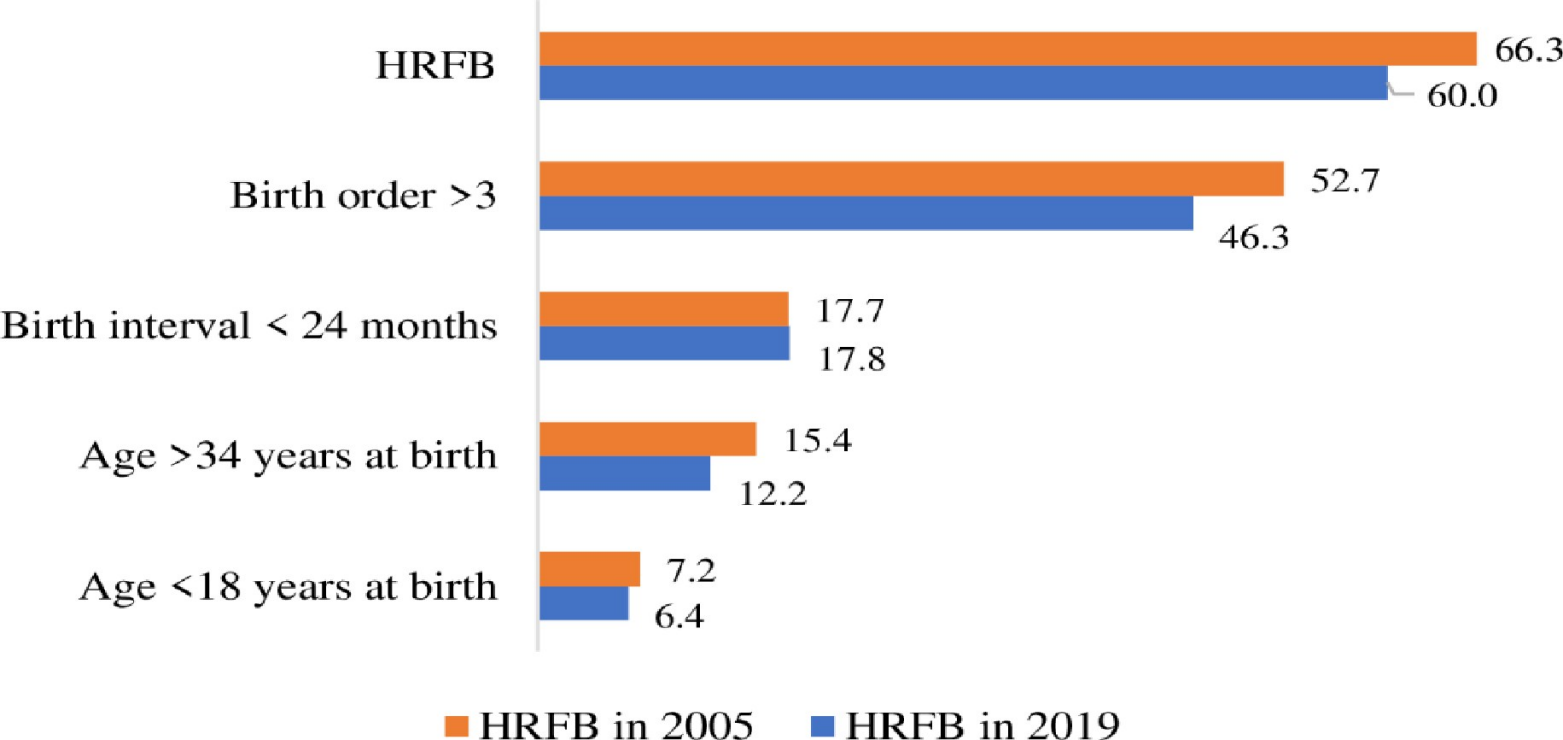
HRFB among women who gave birth in the last five years preceding EDHS 2005 (n = 11,163) and 2019 (n = 5,527), Ethiopia.

The prevalence of avoidable HRFB among women who had given birth within the previous five years before the survey was 66.3% (95% CI: 65.1%, 67.4%) in 2005 and 60.0% (95% CI: 58.0%, 62.0%) in 2019. Of these HRFB, high parities had the highest prevalence (52.7% in 2005 and 46.3% in 2019), whereas age below 18 years at the time of birth had the lowest, at 7.2% in 2005 and 6.4% in 2019 (Fig 2). There was a large gap in HRFB between women with no formal education and those with higher education (39.2% of the absolute difference in 2005 increased to 59.0% in 2019). Moreover, HRFB among women with no formal education increased over time (7.7% of the absolute difference), whereas HRFB decreased among women with higher education over time (-12.1% of the absolute difference). There was a decreasing urban-rural disparity over time in HRFB, with 37% to 17.7% of the absolute difference in 2005 and 2019, respectively. The disparity in HRFB between women from the poorest and richest households increased over time; the absolute difference in HRFB between the poorest and richest was 12.6% in 2005 and increased to 37% in 2019. Moreover, HRFB among women from the richest households was reduced from 56.9% in 2005 to 35.2% in 2019 (Table 2).

**Table 2:** HRFB among women who gave birth within the last five years preceding EDHS 2005 (n = 11,163) and 2019 (n = 5,527), Ethiopia.

Variables	2005		2019	
	HRFB (weighted frequencies n (%))		HRFB (weighted frequencies n (%))	
	Yes	No	Yes	No
Education status				
No education	6,036 (68.3)	2,802 (31.7)	2,250 (76.0)	711(24.0)
Primary	1,184 (63.8)	671(36.2)	924 (47.2)	1,032 (52.8)
Secondary	163 (38.3)	264 (61.7)	108 (26.0)	307 (74.0)
Higher	13 (29.1)	30(71.9)	33 (17.0)	161 (83.0)
Marital status				
Not in union	324 (50.2)	321 (49.8)	171 (56.6)	131 (43.4)
In union	7,072 (67.2)	3,446 (32.8)	3,144 (60.2)	2,080 (39.8)
Sex of household head				
Male	6,654 (66.9)	3,289 (33.1)	2,883 (60.4)	1,893 (39.6)
Female	742 (60.8)	478 (39.2)	433 (57.6)	318 (42.4)
Religion				
Orthodox	2,852 (61.0)	1,822 (39.0)	952 (51.2)	906 (48.8)
Protestant	1,523 (68.7)	694 (29.3)	882 (60.4)	578 (39.6)
Muslim	2,735 (70.6)	1,141 (29.4)	1,406 (66.9)	694 (33.1)
Other	287 (72.2)	110 (27.8)	75 (70.8)	31 (29.2)
Residence				
Rural	7,031 (67.9)	3317 (32.1)	2,678 (64.4)	1,482 (35.6)
Urban	366 (44.9)	449 (55.1)	638 (46.7)	729 (53.3)
Region				
Tigray	431 (61.8)	267 (38.2)	178 (47.8)	194 (52.2)
Amhara	1,611 (61.5)	1,010 (38.5)	544 (51.8)	506 (48.2)
Oromia	3,095 (70.2)	1,316 (29.8)	1,424 (64.4)	787 (35.6)
Somali	337 (70.6)	140 (29.4)	317 (77.5)	92 (22.5)
SNNPR	1,676 (67.0)	824 (33)	679 (61.4)	427 (38.6)
City Administrative	72 (37.7)	119 (62.3)	58 (31.0)	128 (69.0)
Other	175 (65.8)	91 (34.2)	60.6	
Place of birth				
Home	7,142 (67.8)	3,387 (32.2)	2,026 (71.3)	816 (28.7)
Health facility	255 (40.2)	380 (59.9)	1,290 (48.0)	1,395 (52.0)
Sex of the baby				
Male	3,744 (65.4)	1,980 (34.6)	1,680 (59.1)	1,162 (40.9)
Female	3,653 (67.2)	1,787 (32.9)	1,635 (60.9)	1,049 (39.1)
Contraceptive use				
No	6,596 (67.0)	3,257 (33.1)	2,223 (67.1)	1,089 (32.9)
Yes	800 (61.1)	510 (38.9)	1,093 (49.3)	1,123 (50.7)
Wealth index				
Poorest	1,697 (69.5)	743 (30.5)	953 (72.2)	368 (27.8)
Poorer	1,608 (68.3)	747 (31.7)	800 (66.8)	398 (33.2)
Middle	1,630 (65.6)	856 (34.4)	670 (64.2)	274 (35.8)
Richer	1,517 (68.3)	704 (31.7)	538 (56.1)	421(43.9)
Richest	944(56.9)	716 (43.1)	354 (35.2)	651 (64.8)

### Socioeconomic inequality in high-risk fertility behavior

**Fig 3:**
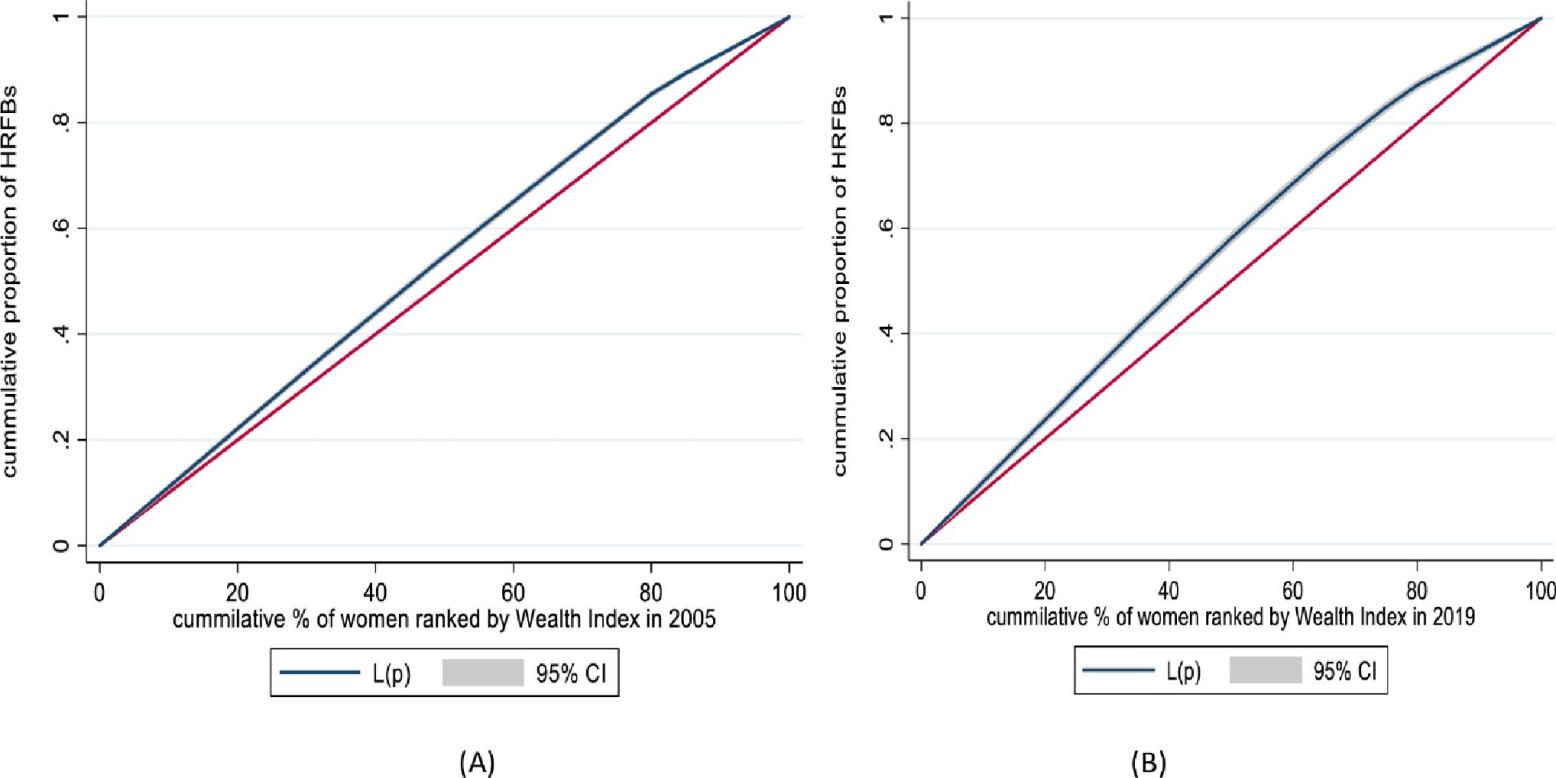
ECI of HRFB among women who had birth within five years preceding EDHS 2005 (A) and 2019 (B), Ethiopia.

Fig 3 illustrates a concentration curve, graphing the cumulative share of HRFB against the proportion of the cumulative wealth index. The weighted ECI analysis result showed that socioeconomic inequality in HRFB widened from -0.0682 (95% CI: -0.1009, -0.0355) with a SE of 0.0167 and a p-value < 0.001 in 2005 to -0.2634 (95% CI: -0.3365, -0.1903) with a SE of 0.0373 and a p-value of <0.001 in 2019. Since the ECI value was negative the concentration curve remained above the line of equality in both surveys, suggesting that HRFB was skewed towards the poor population (pro-poor). To achieve a balanced distribution of HRFB, redistribution measures across the wealth spectrum are crucial. One proposed method for achieving a more equitable redistribution (R) is the linear scheme, which can be calculated using the formula R = CI*75 [41]. When the CI is multiplied by 75, which was 0.0682*75 = 5.1% in 2005 and 0.2634*75 = 19.7% in 2019, it implies that 5% of HRFB in 2005 and 20% of HRFB in 2019 would be redistributed from the poorest half to the richest half of the population to attain a distribution with an index value of zero (Fig 3).

### The erreygers decomposition analysis

As the concentration index and curve results showed socioeconomic-related inequality in HRFB, decomposition analysis was performed to assess and quantify how much of the inequality was explained by the wealth quantiles and other variables. Coefficient, elasticity, ECI, absolute contribution (AC), and percentage contribution (PC) were estimated to better understand the variables contributing to socioeconomic inequality. The ‘Elasticity’ column, a unit-free measure of partial association, reflects how much the dependent variable (in this case, socioeconomic inequality in HRFB) changes in response to a one-unit shift in the explanatory variables. It specifically indicates the sensitivity of socioeconomic inequality in HRFB to changes in the determinant variables. A positive elasticity value suggests an increase in HRFB with a positive change in the determinant, while a negative value indicates a decrease. For example, in the 2005 EDHS, the elasticity for women with primary education was −0.0252, indicating that a 1% change in women’s education from no education to primary education would result in a 2.5% decrease in socioeconomic inequality in HRFB. Similarly, a 1% change in women’s education from no education to secondary education (-2.4%) and higher education (-0.3%) reduced the pro-poor socioeconomic inequality in HRFB. The ’ECI’ column reflects the distribution of determinants itself with wealth quintiles. The positive or negative sign of the ECI reflects whether the determinants of inequality are concentrated among wealthier or poorer households, respectively. For example, in the 2019 EDHS, individuals with primary, secondary, and higher education levels were more likely to be concentrated in the wealthier segment, while Muslim and other religious groups were more commonly found in the lower end of the wealth distribution. The ’percentage contribution’ indicates how much each determinant contributes to overall socioeconomic inequality in HRFB. A positive percentage contribution’ shows that a factor increases the observed inequality, whereas a negative contribution indicates a reduction in the inequality. In this study, educational status contributed the highest percentage to prop-poor inequalities in HRFB, accounting for 10.2% in 2005 and rising to 28.1% in 2019. Place of birth was another predictor that contributed significantly to prop-poor inequalities in HRFB, accounting for 7.0% in 2005 and increasing to 25.6% in 2019. Religion and region contributed to prop-poor inequalities in HRFB declined from 15.9% to 3.6% and 9.1% to 2.7%, respectively. Furthermore, the contribution of wealth index rank and contraceptive use for inequalities increased from -12.9% to 14.5% and -1.5% to 11.6%, respectively, but they were statistically significant only in 2019. The decomposition also suggests that while the model has identified and explained most of the inequality (79.92%), there is still a portion (20.8%) that requires further investigation. This residual may point to the need for more comprehensive data or consideration of additional factors in future research or policy interventions. Addressing the explained portion through targeted policies (e.g., improving education or income equality) may help reduce inequalities, but the residual portion reminds us that inequalities are complex and multifaceted (Table 3).

**Table 3:** Factors contributing to HRFB inequality among women who had birth within the five years preceding EDHS 2005 (n = 11,163) and 2019 (n = 5,527), Ethiopia.

	2005					2019				
Variable	Coefficient	Elasticity	ECI	AC	PC	Coefficient	Elasticity	ECI	AC	PC
Education status (ref.no education)										
Primary	-0.0379***	-0.0252	0.1680	-0.0042	6.20	-0.2324***	-0.3290	0.1541	-0.0507	19.25
Secondary	-0.1593***	-0.0244	0.1107	-0.0027	3.96	-0.3681***	-0.1105	0.1572	-0.0174	6.59
Higher	-0.1699**	-0.0026	0.0131	-0.0001	0.05	-0.4189***	-0.0588	0.1021	-0.0060	2.28
Subtotal					10.21					28.12
Marital status (ref. not in union)										
In union	0.1291	0.4867	-0.0004	-0.0002	0.30	0.0023	0.0085	-0.0254	0.0002	0.08
Sex of household head (ref. Male)										
Female	0.0170	0.0074	-0.0068	-0.0001	0.09	-0.0136	-0.0074	0.0207	-0.0002	0.06
Religion (ref. Orthodox)										
Protestant	0.0517**	0.0411	0.1156	0.0047	-6.96	0.0469***	0.0496	0.0707	0.0035	-1.33
Muslim	0.0442***	0.0614	-0.2501	-0.0154	22.52	0.0413**	0.0628	-0.2048	-0.0129	4.88
Other	0.0720***	0.0102	-0.0196	-0.0002	0.29	0.0169*	0.0013	-0.0227	-0.0001	0.01
Subtotal					15.85					3.56
Residence (ref. Rural)										
Urban	-0.0787	-0.0230	0.2267	-0.0052	7.64	0.0277	0.0274	0.5021	0.0138	-5.22
Region (ref. Tigray)										
Amhara	-0.0232	-0.0217	0.0387	-0.0008	1.23	-0.0488	-0.0371	0.0488	-0.0018	0.68
Oromia	0.0323	0.0510	-0.0727	-0.0037	5.44	0.0433	0.0693	-0.0567	-0.0039	1.49
Somali	0.0253	0.0043	-0.0823	-0.0004	0.52	0.0404*	0.0119	-0.1406	-0.0017	0.64
SNNPR	-0.0104	-0.0093	0.1301	-0.0012	1.77	0.0279	0.0224	0.0239	0.0005	-0.20
City Administrative	-0.0243**	-0.0017	0.0486	-0.0001	0.12	-0.0111	-0.0015	0.0986	-0.0001	0.06
Other	0.0054	0.0005	-0.0145	-0.0001	0.01	0.0021	0.0003	-0.0231	-0.0001	0.01
Subtotal					9.09					2.68
Place of birth (ref. Home)										
Health facility	-0.1560***	-0.0355	0.1336	-0.0047	6.95	-0.0704***	-0.1367	0.4927	-0.0674	25.58
Sex of the baby (ref. Male)										
Female	0.0150	0.0293	0.0043	0.0001	-0.19	0.0264	0.0513	0.0559	0.0029	-1.09
Contraceptive use (ref.no)										
Yes	0.0118	0.0055	0.1792	0.0010	-1.45	-0.0683***	-0.1095	0.2796	-0.0306	11.62
Wealth index (ref. poorest)										
Poorer	-0.0067	-0.0057	-0.2970	0.0017	-2.47	-0.0058*	-0.0050	-0.2647	0.0013	-0.51
Middle	-0.0235	-0.0209	0.0729	-0.0015	2.24	0.0264	0.0120	0.0756	0.0015	-0.57
Richer	0.0136	0.0109	0.4009	0.0044	-6.38	-0.0155	-0.0108	0.3213	-0.0035	1.31
Richest	0.0142	0.0085	0.5063	0.0043	-6.27	-0.0870*	-0.0633	0.5953	-0.0377	14.3
Subtotal					-12.88					14.53
Explained				-0.0244	35.61				-0.2104	79.92
Residual				-0.0438	64.4				-0.0530	20.08

Key: ref = reference

* significant at <0.05

** significant at <0.01

*** significant at <0.001

AC = absolute contribution, PC = percent contribution, and ECI = Erreygers concentration index.

## Discussion

This study aimed to assess the socioeconomic-related inequalities in HRFB in 2005 and 2019 by employing the ECI. This study also identified persistent over time and newly emerging factors of pro-poor inequality in HRFB, which is critical for policymakers and partners to undertake targeted interventions. Furthermore, to our knowledge, this is the first study that looks at socioeconomic inequality in HRFB, which is important to reducing pro-poor inequalities and the burden of HRFB in low- and middle-income countries like Ethiopia. This study’s ECI and curve findings demonstrated that HRFB was disproportionately concentrated among women in lower socioeconomic groups (pro-poor) in both surveys. Moreover, the socioeconomic inequality in HRFB widened over time. It can be attributed to disparities in access to healthcare services and limited economic opportunities exacerbating this inequality [42], as individuals with higher socioeconomic status are better equipped to make informed reproductive decisions and access family planning services [43]. Furthermore, cultural and social norms prevalent in low-income and low-education populations may influence fertility decision-making. These norms may prioritize early marriage, and large family sizes, particularly in rural and traditional societies. Societal norms influence women’s HRFB, as they face cultural pressures to have more children or prefer male offspring, which pushes them towards HRFB. Then, women’s inner fear that if they do not fulfill societal or family demands, their husbands/partners will go for polygamy/second marriage also results in HRFB among women [44].

The decomposition analysis showed that educational status had significantly contributed to socioeconomic inequalities in HRFB and persisted over time. Previous studies have supported the current findings, revealing that women with little or no education [3, 45] are more likely to experience HRFB. Individuals with lower educational status often face challenges in accessing healthcare services and making informed decisions about their health, including reproductive health [30]. This lack of access and information can result in limited preconception counseling and family planning education, leading to lower contraceptive use and higher rates of HRFB. Moreover, those who lack information about reproductive health and services may not understand well the importance of spacing in pregnancies, limiting the number of births, and the risks associated with early or late-age pregnancies [46]. Economic disparities may further exacerbate this situation by hindering individuals’ ability to afford transportation and other costs related to accessing healthcare services and information through mass media [47].

In this study, wealth index rank and contraceptive use emerged as new factors that explain the pro-poor inequality HRFB in 2019. This shift could be attributed to economic improvement and increased availability of family planning services over time [7, 48]. Disparities in educational opportunities and economic growth contribute to differences in contraceptive uptake, with economically and educationally disadvantaged women often having lower rates of contraceptive use [49, 50]. Moreover, early marriage is often prevalent among economically and educationally disadvantaged women in Ethiopia [51]. This can result in early childbirth, further deepening societal inequalities. Conversely, in 2005, lower socioeconomic status and limited access to healthcare services likely hindered effective family planning, resulting in a weaker correlation between wealth index rank, contraceptive use, and HRFB [37]. Moreover, lack of empowerment among women as most of the males were the family head also contributed to the low rate of contraceptive use. Furthermore, religious beliefs that are more prevalent among the rural population as most of the women resided in rural areas in 2005, are also one of the contributing factors to the low rate of contraceptive use.

In this study, the place of birth played a crucial role in contributing to inequalities in HRFB and persisted over time. This finding was supported by a previous study [38]. Health facility delivery reflects women’s access to healthcare facilities. Limited access to healthcare facilities in rural Ethiopia drives reliance on home births due to geographical barriers and financial constraints [52]. Low socioeconomic status hampers maternal health service uptake, limiting access to reproductive information and contraceptive utilization. Additionally, cultural preferences for traditional birth attendants over formal healthcare providers exacerbate the situation, further hindering informed reproductive decisions among women [32]. Home delivery in Ethiopia correlates with neonatal mortality, and the loss of newborns can lead women to engage in high-risk fertility behavior, driven by the desire to replace those lost and the fear of death. This cycle causes the women to engage in risk fertility behavior, emphasizing the urgent need for improved maternal healthcare services and education [9].

Our study also showed that the contribution of religion and region in HRFB inequalities was notable and persisted over time. Previous studies also documented the disparities of HRFB by religion and region [18, 22, 53]. Criticism toward contraceptive use coexists with religious encouragement of early marriage in some religions [54]. Furthermore, disparities in healthcare access and education persist across regions in Ethiopia, particularly developing regions where reproductive healthcare services are often inadequate, and literacy rates are lower. Regions with higher levels of economic prosperity and better access to healthcare facilities may exhibit lower levels of socioeconomic inequality in HRFB compared to regions with limited resources and infrastructure. Evidence has shown that poverty and illiteracy are widespread and severe among emerging and pastoralist regions in Ethiopia due to their scattered and nomadic lifestyle [55]. These factors collectively impede access to family planning counseling and information, thereby contributing to high-risk fertility behaviors.

This study utilized nationally representative data from the 2005 and 2019 EDHS, making its findings paramount. It revealed socioeconomic disparities in HRFB over time and identified the underlying factors. These results allow policymakers to improve the fair delivery of counseling, information, and services to address the observed HRFB inequalities by focusing on these identified factors. Nevertheless, this study may encounter certain limitations. The decomposition analysis does not establish a cause-and-effect relationship between exploratory and outcome variables; rather, it predominantly signifies correlation. Socioeconomic status and HRFB were evaluated based on self-reports from participants, potentially introducing subjective bias. Moreover, the assets-based wealth index, used as a proxy for household economic status, may only sometimes provide accurate results compared to direct measurements of income and expenditure where such data are available or can be collected reliably. Furthermore, some crucial variables–household income, expenditure, political economy, availability of youth-friendly health services, access to health care, timing to contraceptive use after sexual debut, and spousal support are absent from this analysis because they need to be collected from the primary source.

## Conclusions

A significant portion of HRFB was observed among women from lower socioeconomic status, and this gap has widened over time. Educational status, place of birth, region, and religion have all played substantial roles in the observed socioeconomic disparities in HRFB and persisted over time. In the latest survey, wealth index rank and contraceptive utilization have emerged as additional significant factors contributing to inequalities. Hence, implementing a pro-poor policy is vital to reducing HRFB among economically disadvantaged segments by addressing social determinants. Designing strategies that guarantee all women have access to and use reproductive health services, coupled with efforts to enhance economic empowerment and educational opportunities, can contribute to mitigating disparities in HRFB. Improving women’s education reduces early marriage, promotes contraception use, and lowers high-risk fertility behaviors.
